# Drinking Water Supplementation of *trans*-Cinnamaldehyde-Miglyol Microemulsions Reduces Multidrug-Resistant *Salmonella* Heidelberg in Turkey Poults and Augments the Antibacterial Effect of Oxytetracycline

**DOI:** 10.3390/microorganisms13122703

**Published:** 2025-11-27

**Authors:** Divek V. T. Nair, Anup Kollanoor Johny

**Affiliations:** Department of Animal Science, University of Minnesota–Twin Cities, Saint Paul, MN 55108, USA; divekvt@gmail.com

**Keywords:** *trans*-cinnamaldehyde, oxytetracycline, *Salmonella*, augmentation, antibacterial

## Abstract

The use of clinically important antibiotics in U.S. poultry production has decreased drastically over the past decade. They can only be used to treat diseases under the supervision of a veterinarian. Reducing antibiotic use, even for disease treatment, can improve the long-term sustainability of the industry. In the current study, we examined the effect of supplementation of a low dose of *trans*-cinnamaldehyde (**TC**; 0.03%), a GRAS-status plant-derived compound, with or without oxytetracycline (**OTC**; 16 μg/mL), an anti-30S ribosomal subunit targeting antibiotic, on the multidrug-resistant (**MDR**) *S.* Heidelberg (**SH**) in turkey poults. Two independent experiments were conducted (N = 96). In each experiment, 48, straight-run, day-old, commercial Hybrid Converter turkey poults were randomly assigned to 6 treatments of 8 birds each: Negative Control [**NC**; −SH, −TC, −OTC, −0.06% Miglyol (**MIG**, emulsifier for TC in water)], Positive Control (**PC**; +SH, −TC, −OTC, −MIG), MIG Control (MIG; +SH, −TC, −OTC, +MIG), TC Group (**TC**; +SH, +TC, −OTC, +MIG), OTC group (**OTC**; +SH, −TC, +OTC, −MIG), and TC+OTC group (**TC+OTC**; +SH, +TC, +OTC, +MIG). OTC was supplemented from day 1 through drinking water throughout the experiment. The birds in the TC and TC+OTC groups were supplemented with TC in their drinking water for 7 days post-challenge. All birds were challenged on day 7 with 6 log_10_ CFU of SH/bird via crop gavage. On day 14, all birds were euthanized to collect the cecum, liver, and spleen for pathogen recovery. TC at 0.03% emulsified in MIG was highly effective in reducing MDR SH colonization in turkey poults (*p* < 0.05) compared to the SH control (>4.5 log_10_ CFU/g reduction) on day 14. The OTC group reduced the pathogen load by 2.5 log_10_ CFU/g by day 14. TC enhanced the effect of OTC, reducing pathogen load by ~3.9 log_10_ CFU/g compared to the SH control after 7 days. TC significantly reduced SH invasion into the liver and spleen compared with the SH control on day 14. The results of the study indicate that TC at 0.03% can augment OTC at 16 μg/mL for the treatment of MDR SH infection in poults and could be an industry-sustainable strategy.

## Simple Summary

Antibiotic use in the U.S. poultry industry has dropped significantly over the last decade and is limited to treating illness under a veterinarian’s care. To support this change, we are exploring sustainable solutions that can fight disease by reducing antibiotic use. In this study, we tested emulsified *trans*-cinnamaldehyde (TC)—a safe natural compound found in cinnamon—to see if it could help control multidrug-resistant *Salmonella*, a harmful bacterium in turkeys. Turkey chicks were divided into groups and given either TC, a common antibiotic called oxytetracycline (OTC), both, or neither. All the birds were infected with *Salmonella*, and a week later, we measured the bacterial load in their organs. The group that received only TC showed the most significant decrease in bacteria. The antibiotic alone also helped, but not as effectively as TC. TC augments OTC, but not synergistically as expected. The research demonstrates that a small dose of emulsified TC could be used to reduce harmful bacteria in turkeys and promote prudent antibiotic use in agriculture, thereby improving the industry’s sustainability.

## 1. Introduction

The annual occurrence of human non-typhoidal salmonellosis in the United States is approximately 1.3 million. Among these, approximately 1 million *Salmonella* infections are attributed to the consumption of *Salmonella*-contaminated food [1]. Poultry, including turkeys, are natural reservoirs of *Salmonella* and a constant source of infection through fecal shedding, resulting from *Salmonella* colonization in the ceca [2]. Pathogen colonization leads to contamination of the surrounding farm environment and contamination of poultry meat during processing [3,4,5]. This situation is a bottleneck to the sustainable advancement of the industry.

*Salmonella* Heidelberg (**SH**) is a highly invasive *Salmonella* serovar that frequently infects humans and is often isolated from poultry production and processing plants. SH can colonize within the cecum of poultry and be transmitted to humans through contaminated poultry [5,6]. SH is among the top five *Salmonella* serotypes causing clinical infections in humans [7] and has been linked to foodborne outbreaks through the consumption of contaminated ground turkey, chicken carcasses, and mechanically separated chicken meat in the US [5]. The development of antibiotic resistance in SH, high invasive capability, and association with foodborne outbreaks through poultry are serious concerns in the U.S. SH isolated from ground turkey and chicken carcasses implicated in outbreaks were resistant to multiple drugs, including the drug of choice for human salmonellosis—ceftriaxone, a third-generation cephalosporin with high activity against Gram-negative organisms [8,9].

With grave concerns about the development of antibiotic resistance among various pathogens, including the emerging MDR *Salmonella*, the FDA introduced the Veterinary Feed Directive (**VFD**), which prohibits the use of prophylactic antibiotics and necessitates veterinary oversight before using clinically important antibiotics in treating production animals [10]. This move underscores the importance of judicious antibiotic use in animal agriculture and emphasizes the need to develop natural, safe, and sustainable intervention strategies against deadly foodborne pathogens, such as *Salmonella*.

The use of natural alternatives against pathogens has garnered interest over the past two decades due to concerns about the development of antibiotic resistance in bacteria. Essential oils have been traditionally used to preserve foods and enhance their flavor [1,3]. The antimicrobial properties of several essential oils have been demonstrated, and a variety of active components have been identified. *trans*-cinnamaldehyde (TC) is the major component of cinnamon. It is classified as GRAS (generally recognized as safe) and is approved for use in foods by the Food and Drug Administration [11,12,13]. Previous studies revealed that TC is effective against *S.* Enteritidis in broilers [11,12,13].

Although antibiotic use in US poultry production has decreased sharply over the past decade, antibiotics should still be used judiciously under veterinary supervision to address animal health concerns [10,14]. These medically important antibiotic drugs (**MIAD**) include oxytetracycline (**OTC**), avilamycin, chlortetracycline, erythromycin, lincomycin, hygromycin B, sulfadimethoxine, sulfaquinoxaline, and virginiamycin. OTC can be effective against a range of pathogens in chickens and turkeys when combined with neomycin and certain ionophore antimicrobials added through dry animal feed [14,15]. Veterinarians can determine the most effective antibiotic for an animal health condition and administer it through injection in a hatchery or mix it into feed or drinking water [10]. Commercially available water-soluble OTC can be given to chickens and turkeys independently for up to 14 consecutive days against various bacterial organisms susceptible to the antibiotic with a required veterinary prescription.

In most studies, TC was supplemented through feed and has not been tested in water due to its limited solubility [11,12,13]. We recently reported that the direct addition of 0.08% TC to poultry drinking water resulted in a significant reduction in SH [1]. However, poor solubility of TC in water was identified as a hindrance to achieving better SH reductions. The role of miglyol (**MIG**), a blend of decanoyl and octanoyl glyceride-based emulsifier, was evaluated in the production of TC-MIG microemulsions. Moreover, the efficacy of plant-derived antimicrobials (**PDA**s), including TC, in augmenting MIADs, such as OTC, has not been studied. If found effective, TC could be a veterinarian’s aid in reducing the use of MIADs during poultry disease treatment, enhancing the sustainability of industry treatment options. Therefore, the objective of the study was to determine the anti-SH efficacy of a low dose of TC (0.03% *v*/*v*) emulsified in MIG (0.06%) alone or in combination with a low dose of OTC (16 μg/mL), administered through water, in turkey poults.

## 2. Materials and Methods

### 2.1. Ethics Statement

The animal studies were approved by the Institutional Animal Care and Use Committee [IACUC#1403-31368A], and the use of infectious agents was approved by the Institutional Biosafety Committee [IBC# 1403-31359H] at the University of Minnesota.

### 2.2. Bacterial Strain and Culture Conditions

The *Salmonella* Heidelberg strain GT2011 (**SH**), which was involved in the 2011 ground turkey outbreak in the U.S., was used in this study to inoculate poults [16,17,18,19,20,21,22,23,24]. The strain has been characterized for its capacity to attach firmly to turkey skin [16], multiplication in cecal contents, attachment to avian intestinal epithelial cells and motility [17], colonization in turkey poults [1,2,20], ability to invade and dissemination to the liver, spleen and skeletal muscles in turkeys [2], and survival in poultry drinking water [1]. The working cultures of SH in tryptic soy broth (**TSB**; catalog no. C7142, Criterion, Hardy Diagnostics, Santa Maria, CA, USA) were prepared from glycerol stocks stored at −80 °C. Then, SH were made resistant to 50 µg/mL nalidixic acid sodium salt (**NA**; CAS. no. 3374-05-8, Alfa Aesar, Haverhill, MA, USA) by continuous subculturing in NA for selective identification of SH strain from the cecum of poults. The growth of NA-resistant SH in overnight broth cultures (16 h) was determined by plating appropriate dilutions of SH on xylose lysine desoxycholate (**XLD**; catalog no. C7322, Criterion, Hardy Diagnostics, Santa Maria, CA, USA) agar plates containing 50 µg/mL NA. To inoculate the poults, the NA-resistant SH were grown in 100 mL TSB, and subcultures were performed every 16 h for 3 days by transferring 1 mL of inoculum into 100 mL TSB containing NA. After three subcultures, the overnight broth cultures containing 10^8^ CFU/mL NA-resistant SH were centrifuged (3600× *g*, 15 min, 4 °C; Allegra X-15 benchtop centrifuge, Beckman Coulter Inc., Fullerton, CA, USA), and the pellet was diluted to 10^6^ CFU/mL in PBS. Then, 2 mL of the suspension was used to inoculate 7-day-old poults via crop gavage [16,17,18,19,20,21,22,23,24].

### 2.3. Plant-Derived Antimicrobial (PDA)

*trans*-cinnamaldehyde (TC) (≥99%; Natural, Food Grade; Catalog # W290106-100G-K; Lot# MKBS7421V) was purchased from Sigma–Aldrich (PO Box 14508, St. Louis, MO, USA). A lower concentration of TC [0.03% (*v*/*v*)] was used in the experiment to supplement the poults through drinking water. This concentration was selected based on our previous experiments. For instance, our group recently reported the use of TC at 0.08% without solvent, delivered via drinking water to poults, resulting in anti-SH effects and a significant difference in the cecal metabolome [1,23]. Previous in vitro experiments have found that TC at a concentration as low as 0.02% could increase the sensitivity of a drug-resistant serotype, *S*. Typhimurium DT104, to tetracycline [24]. In this study, TC was dispersed in water using an inert diluent, miglyol (**MIG**; 0.06% *v*/*v*; IOI Oleo GmbH, D-58453 Witten, Germany). In our experiments, we found that 0.02% TC and 0.06% MIG interacted to produce emulsions effective against SH, while 0.06% MIG alone showed no antibacterial effects. Water containing TC and MIG was thoroughly mixed before being provided to poults. The particle size distribution of the treatments was determined using a laser diffraction particle size analyzer (Bluewave, Microtrac Inc., Montgomeryville, PA, USA) at the Minnesota Nano Center.

### 2.4. Oxytetracycline (OTC)

A pure form of OTC hydrochloride (O5875, Sigma Aldrich Inc., St. Louis, MO 63103, USA; https://www.sigmaaldrich.com/specification-sheets/350/909/O5875-10G.pdf, accessed on 19 November 2025) at the sub-therapeutic dose of 16 µg/mL was used to supplement the poults from day 1 to 14 through drinking water. OTC is a tetracycline analog isolated from *Streptomyces rimosus*, used to treat infections caused by both Gram-positive and Gram-negative microorganisms. OTC is lipophilic, so it can easily pass through the bacterial cell membrane. The concentration tested in this study (16 µg/mL) was selected based on our previous study, which determined the subinhibitory concentration of tetracycline against *S*. Typhimurium DT104 [24]. Furthermore, this concentration was found to be more than 6 times lower than the highest concentration of commercial OTC products recommended by multiple manufacturers against various disease-causing microorganisms in poultry (e.g., Pennox 343^®^ by Pharmgate Animal Health, Wilmington, NC, USA; https://pharmgate.com/usa/wp-content/uploads/sites/5/2025/08/PAH_Pennox343_Product-Profile_21133-0825.pdf, accessed on 19 November 2025).

### 2.5. Experimental Design and Animal Management

Two experiments were conducted. In each experiment, 48 straight-run, day-old, commercial Hybrid Converter turkey poults were randomly assigned to 6 treatments (8 birds/group). Each treatment group was housed in separate Biosafety Level–2 (BSL2) isolators in the Research Animal Resources (RAR) facilities at the University of Minnesota. The birds were supplied with *Salmonella*-free ad libitum feed (Famo Feeds Inc., Freeport, MN, USA), water, space, and light appropriate to the age group of the poult. The six treatments included in each experiments were: (1) Negative Control [**NC**; −SH, −TC (0.03%), −OTC, −0.06% Miglyol (MIG, diluent)], (2) Positive Control (**PC**; +SH, −TC, −OTC, −MIG), (3) MIG Control (**MIG**; +SH, −TC, −OTC, +MIG), (4) TC Group (**TC**; +SH, +TC, −OTC, +MIG), (5) OTC group (**OTC**; +SH, −TC, +OTC, −MIG), and (6) TC+OTC group (**TC+OTC**; +SH, +TC, +OTC, +MIG). OTC was supplemented from day 1 through drinking water throughout the experiment. All poults except those in NC were challenged on day 7 with 6 log_10_ CFU of SH through crop gavage. The birds in the TC and TC+OTC groups were supplemented with TC (0.03%) via drinking water from day 8 to day 14. On day 14, all poults were euthanized to collect cecum, liver, and spleen for SH recovery and microbiological analysis.

### 2.6. Determination of S. Heidelberg in the Cecum, Liver, and Spleen of Turkey Poults

On day 14 (7 days post-challenge), samples were collected from all the poults to determine the effects of different treatments on SH colonization in the cecum and its dissemination to the liver and spleen. The cecum with its contents, liver, and spleen were collected separately in 10 mL PBS and homogenized. The cecal samples were 10-fold serially diluted in PBS, and 200 µL of the appropriate dilutions were plated on XLD+NA plates. The plates were incubated at 37 °C for 48 h for SH enumeration. The homogenized liver and spleen samples were enriched with 10 mL selenite-cysteine broth (**SCB**; Catalog no. C6921, Criterion, Hardy Diagnostics, Santa Maria, CA, USA) and incubated at 37 °C. After 10 h of incubation, the enriched samples were streaked onto XLD+NA plates and incubated for 24 h at 37 °C for SH recovery. In addition, cecal samples were enriched using the same procedure when they were negative for SH by direct plating [16,17,18,19,20,21,22,23,24].

### 2.7. Statistical Analysis

A completely randomized design was employed for the experiments. Each poult was considered an experimental unit, and the samples were collected from individual poults. Datasets were logarithmically (log_10_) transformed before analysis. Analysis of variance tests were performed using the PROC-MIXED procedure of SAS 9.4. Treatment means were separated using Tukey’s Studentized Range Test. The Wilcoxon Rank-Sum test was used to analyze the presence/absence of SH in liver and spleen samples. The treatments were found to be significantly different from the control at *p* value < 0.05 [1,3,4,25].

## 3. Results

**Figure 1:**
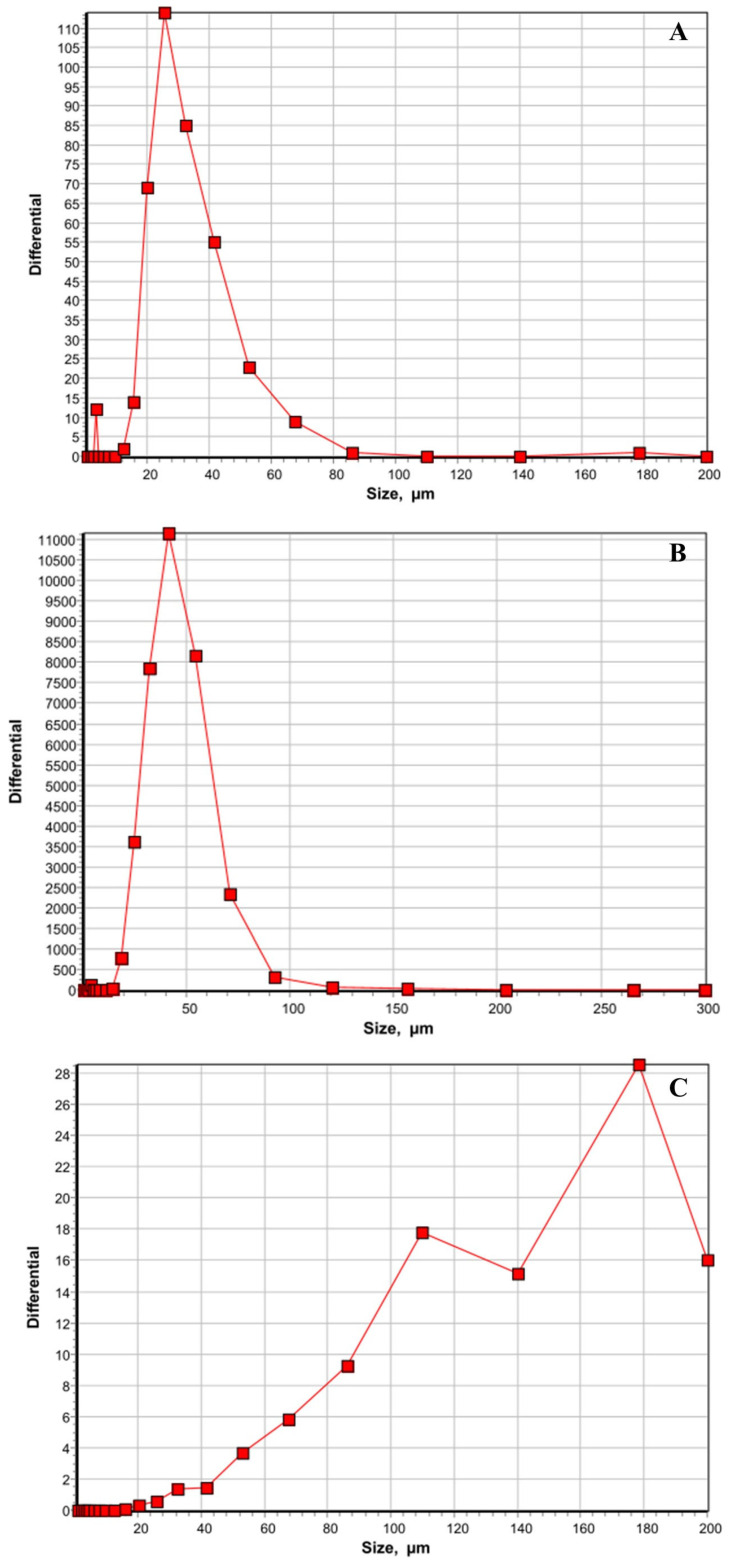

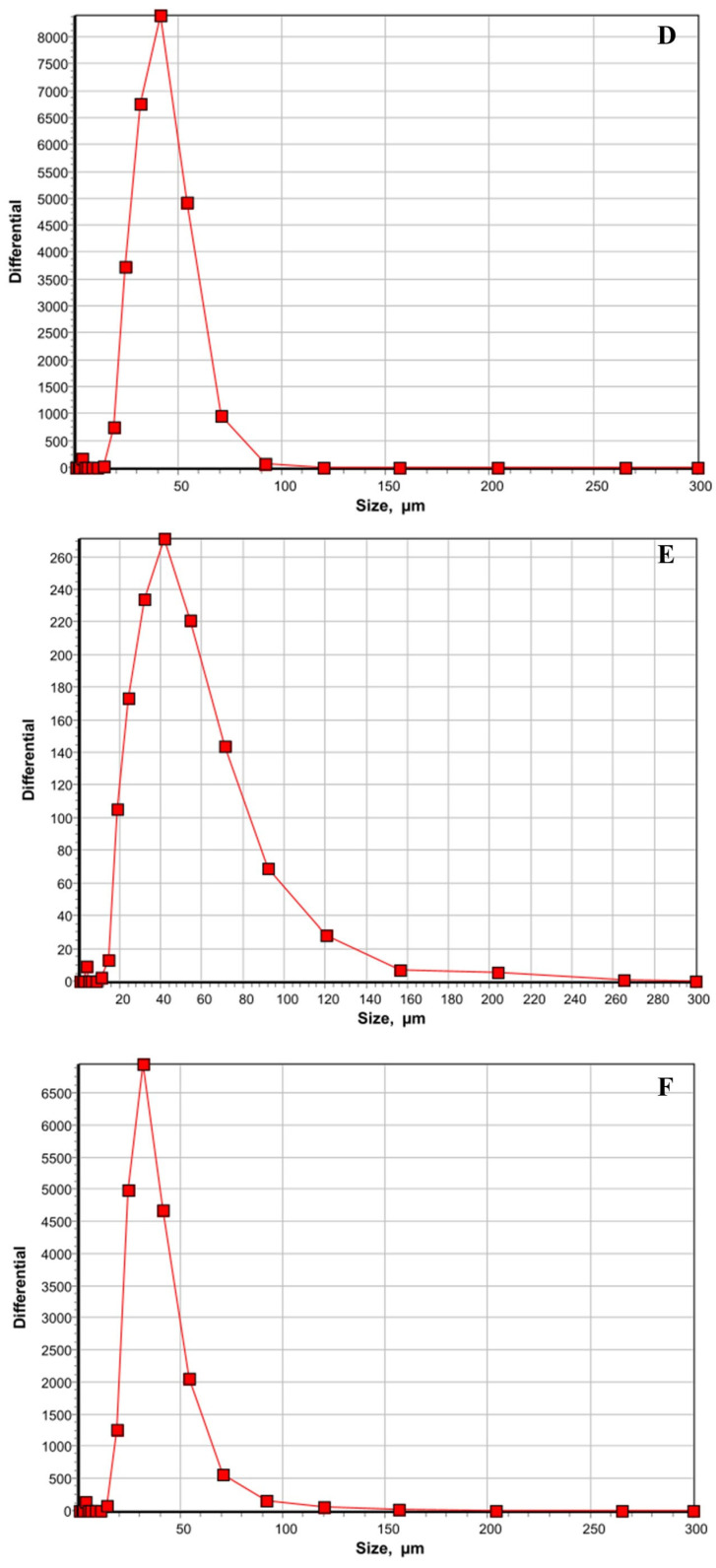
(**A**–**F**): Determination of the particle size distributions of the treatments using a laser diffraction particle size analyzer. All the treatments were prepared in water, and the *trans*-cinnamaldehyde (TC) was dispersed in water using miglyol (MIG). Oxytetracycline (OTC) was directly added to the water. After 30 s of vortexing, the particle size was determined. The six treatments included were plain water (**A**), MIG (**B**), TC (**C**), TC+MIG (**D**), OTC (**E**), and TC+MIG+OTC (**F**). The size of the nanoparticles in µm is plotted on the X axis, whereas the differential is plotted on the Y axis.

The particle size distributions of the treatments were determined using a laser diffraction particle size analyzer to evaluate the dispersion ability of MIG in poultry drinking water (Figure 1A–F). The laser diffraction analysis revealed that the particles in the water ranged from 20 to 80 µm, with a median particle size of 28 µm and a peak size (mode) of 23 µm (Figure 1A). Since the water was unfiltered, these particles represent the particles typically present in the city’s tap water supply. Addition of MIG to water showed a median particle size of 41–50 µm and a peak abundance at ~36 µm (Figure 1B). A wide particle size distribution was obtained when TC was added to the water, ranging from 20 to 200 µm. The mean particle size was ~140 µm, consistent with the largely visible droplet formation when TC was added to the water (Figure 1C). When TC was mixed with MIG in water, particle size decreased compared to TC alone, with a medium droplet size of 42 µm (Figure 1D). This result indicates that MIG can disperse TC more readily in water than TC alone, confirming our observations from visual examination. The combination had particle distributions slightly higher than those of water, but closer to MIG in water. As expected, OTC possessed high solubility in water, and the particle size distribution was similar to that of the tap water (Figure 1E). The size distribution of particles when TC was mixed with MIG and OTC was identical to that of TC and MIG together, which also indicates the high dispersing capability of MIG (Figure 1F).

**Figure 2:**
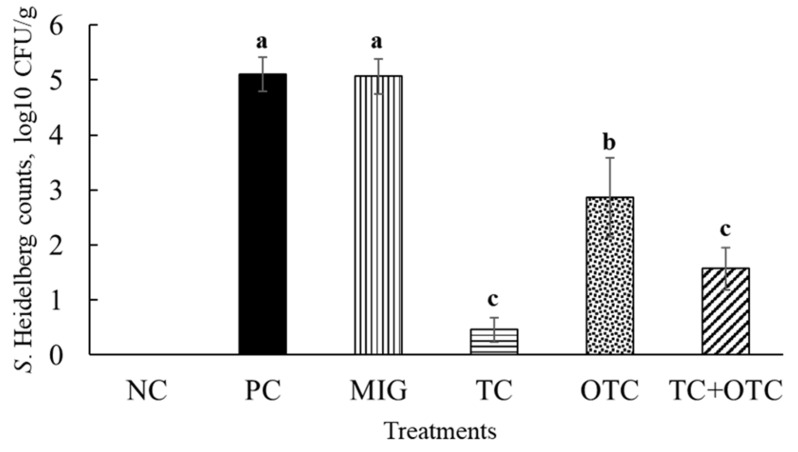
Effects of Treatments on SH Colonization in Poultry Cecum. The six treatments included in each experiments were: (1) Negative Control [NC; −SH, −TC (0.03%), −OTC, −0.06% Miglyol (MIG, emulsifier)], (2) Positive Control (PC; +SH, −TC, −OTC, −MIG), (3) MIG Control (MIG; +SH, −TC, −OTC, +MIG), (4) TC Group (TC; +SH, +TC, −OTC, +MIG), (5) OTC group (OTC; +SH, −TC, +OTC, −MIG), and (6) TC+OTC group (TC+OTC; +SH, +TC, +OTC, +MIG). OTC was supplemented from day 1 through drinking water throughout the experiment. All poults except those in NC were challenged on day 7 with 6 log_10_ CFU of SH through crop gavage. The birds in the TC and TC+OTC groups were supplemented with TC (0.03%) via drinking water from day 8 to day 14. On day 14, all the poults were euthanized to collect cecum, liver, and spleen for SH recovery and microbiological analysis. a, b, c—Bars with different superscripts are significantly different from each other at *p* ≤ 0.05.

The microbiology results revealed that SH colonized to ~5 log_10_ CFU/g in the cecum of turkey poults after 7 days of inoculation (14-day-old turkeys) (Figure 2). This strain of SH has been previously reported to colonize poult ceca efficiently in various experimental challenge studies [1,2,20]. Adding MIG to drinking water yielded SH counts comparable to those in PC, indicating that MIG alone (0.06%) was not bactericidal or bacteriostatic. The therapeutic supplementation of 0.03% TC (solubilized in MIG) was highly effective in poults against SH, which resulted in a >4.5 log_10_ CFU/g reduction in SH in the cecum compared to PC (Figure 2). The OTC was found to be less effective in poults, resulting in a ~2 log_10_ CFU/mL reduction in SH populations compared to PC when added directly. In contrast, TC+OTC resulted in a greater decrease in SH in the cecum than OTC alone, but a lower reduction than TC alone.

The TC, OTC, and TC+OTC treatments significantly reduced SH dissemination to the liver and spleen (Table 1). In our study, 56% liver samples in PC were positive for SH. However, 6%, 0%, and 6% of the liver samples were positive for SH in TC, OTC, and TC+OTC treatments, respectively (*p* < 0.05). Similarly, 38% of the spleen samples from turkey poults in PC were positive for SH, whereas only 6%, 13%, and 0% were positive for SH dissemination (*p* < 0.05). However, the effectiveness of TC, OTC, and TC+OTC treatments for SH dissemination to internal organs was not significantly different among them (*p* > 0.05) (Table 1).

## 4. Discussion

The emergence of antibiotic resistance in foodborne pathogens is a significant concern for both human and veterinary public health, as it increases the economic cost of controlling such pathogens and threatens the sustainability of animal production systems. In light of this, the U.S. FDA implemented stringent measures to prevent the emergence of antimicrobial resistance, particularly by governing the use of antibiotics in poultry production. Most prominent among them was the finalization of VFD, which requires veterinary oversight of the use of medically important antimicrobials in feed and water and limits their use to therapeutic purposes under veterinary care [10].

There has been a significant decline in the use of key antibiotics historically used to control *Salmonella* infections in the United States. For example, in-feed tetracycline use dropped from near-universal use (~90%) in 2013 to effectively 0% since 2019. Tetracyclines, previously used extensively for growth promotion and prophylactic disease prevention, have been reassigned under the VFD and are now legally utilized only for therapeutic indications in poultry and other food animals. Historically, tetracyclines have accounted for the most significant volume of antibiotic use in animal agriculture. However, recent industry data indicate a continued reduction in their use. From 2016 to 2023, total tetracycline use in poultry decreased significantly, mirroring the overall industry trend away from subtherapeutic uses. The use of another vital antibiotic, virginiamycin, plummeted by approximately 99% from 2013 to 2023. Additionally, the use of gentamicin in hatcheries has decreased notably (48%) from 2013 to 2023, with VFD oversight curtailing routine prophylactic use against *Salmonella* [10].

Only some VFD-labeled combinations are currently approved for poultry, further restricting their application. A few MIADs commonly used for *Salmonella* control have been subject to heightened oversight. For instance, gentamicin, an aminoglycoside antibiotic used for years in hatchery injections to prevent early *Salmonella* infections, has VFD-approved status, must be prescribed by a veterinarian, and is restricted in use, leading to significant reductions in hatchery-use reports. Chlortetracycline (CTC), a former antibiotic of choice for *Salmonella* control in layer and broiler feed, transitioned from over-the-counter to VFD status, drastically reducing its routine use [10]. The Food Animal Residue Avoidance Databank (**FARAD**) report [15] describes how some antibiotic combinations have transitioned from broad applications to treatment-only strategies under VFD guidelines. These transitions reflect an industry-wide commitment to antibiotic stewardship and the adoption of alternative approaches such as vaccines, probiotics, and biosecurity enhancements to treat bacterial infections and reduce pathogen carriage within flocks [14].

Among the antibiotic alternatives, extensive research is being conducted on PDAs against foodborne pathogens, given their multiple mechanisms of action. This property of PDAs considerably reduces the possibility of developing antibiotic resistance to them. In the present study, we used TC as an alternative therapeutic intervention to prevent colonization by non-typhoidal SH in turkey poults. A significant issue with TC is its low solubility in water. We have previously conducted research on the direct use of TC in the feed of broiler and layer chickens without any adverse effects on their body weights. In these studies, higher concentrations of TC (0.75% and 1%) were required to reduce *S*. Enteritidis in broiler chickens and layers [11,12,13]. To reduce effective TC concentrations and improve outcomes, we conducted studies evaluating TC supplementation via water in turkey poults infected with SH. We found that the direct addition of 0.08% TC to turkey drinking water resulted in a marked reduction in SH, accompanied by significant changes in metabolites detected using untargeted metabolomics [1,25]. In the current study, our objective was to further reduce TC concentration in poultry drinking water by emulsifying it with MIG, a blend of medium-chain triglycerides (caprylic and capric) derived from plant sources such as coconut and palm kernel oils.

We found that emulsified TC alone or in combination with OTC was highly effective for SH control in turkey poults (Figure 2; Table 1), indicating that TC was more available when emulsified with MIG against the pathogen. The results revealed that TC was more efficacious than OTC treatment. In a more recent study, we reported that TC at 0.08% added directly to poultry drinking water resulted in a significant reduction in SH populations in turkey poults [1]. However, the reduction observed (1.2 log_10_ CFU/g) was lower in magnitude than that observed in the current study (>4.5 log_10_ CFU/g). Dewi and co-workers [1] supplemented TC directly and on alternate days throughout the study, even before the challenge was performed in the poults. Similarly, this study found that TC and TC+OTC were highly effective in reducing SH dissemination to the liver and spleen, compared with the previous research [1]. This discrepancy could be attributed to the low solubility of TC in poultry drinking water, which leads it to settle to the bottom of the waterer, and to the potential absence of continuous SH suppression due to its provision on an alternate-day basis. To circumvent those challenges, we adopted constant supplementation with TC for 7 days post-SH challenge and a TC-MIG combination, resulting in increased TC intake by the poults.

Previous studies on TC supplementation via feed showed that prophylactic supplementation of 0.5% and 0.75% TC resulted in a consistent > 3 log_10_ CFU/g reduction in *S.* Enteritidis in 20-day-old broiler chickens when the birds were challenged at 8 days old with ~8.0 log_10_ CFU *S.* Enteritidis [11]. Additionally, therapeutic supplementation of 0.75% TC for 5 days before slaughter at market age (42 days) resulted in a 1.5 and 2.0 log_10_ CFU/g reduction in *S.* Enteritidis in the cecum and cloaca, respectively, when the broilers were challenged with 8.0 log_10_ CFU *S.* Enteritidis at 30 days old [12]. TC supplementation in feed was also found to be effective in reducing its colonization in the cecum, liver, and oviduct of layers and subsequently reduced *S.* Enteritidis persistence in eggshell and in yolk when supplemented with 1% or 1.5% TC (vol/wt.) for 66 days in 40- or 25-week-old layers [26]. The TC concentrations in the feed are higher than those in water supplementation. The TC-MIG microemulsions reduced SH to >4.5 log_10_ CFU/g (Figure 2, Table 1), a greater reduction than reported in previous studies [11,12,27]. However, this finding needs to be validated in adult turkeys.

The PDAs mainly target the bacterial cell membranes. These compounds disrupt cell membranes, deplete intracellular ATP, and impair enzyme systems, leading to the leakage of cellular contents. The antimicrobial activity of TC is attributed to its conjugated double bonds and the long methyl side chain outside the ring structure [28]. It is found to be highly effective against *S.* Enteritidis at its subinhibitory concentrations. It inhibits transcription of membrane proteins and regulates genes in *Salmonella* Pathogenicity Island I and those associated with invasion of intestinal epithelial cells. Additionally, TC downregulates the genes related to flagellar motility [13]. Additionally, TC affects virulence genes responsible for its colonization of the oviduct [26]. Dewi and co-workers [23] reported that TC treatment increased the abundance of hydroxybutyric acids, which are enhanced by the gut microbiota. In addition, TC was also found to decrease serotonin levels, which could be enriched when enteric pathogens such as *Salmonella* are abundant in the gut [27]. It has previously been reported that the antimicrobial effect of TC could be more pronounced against coliforms than against lactic acid bacteria [29]. In another study, Dewi and co-workers [23] found higher levels of lactic acid in TC-treated birds than in SH controls, suggesting that TC treatment could potentially disrupt the *Salmonella* outer membrane. These findings indicate that TC could function in multiple ways against SH, even when applied alone.

Tetracyclines and their derivatives are effective treatments against *Salmonella* in poultry. Simultaneous administration of chlortetracycline with the *S.* Enteritidis challenge during the first week of hatch reduced its cecal colonization by 5 log_10_ CFU/g in chicks. This is followed by the complete elimination of *S*. Enteritidis in the second week, with continuous antibiotic administration at 200 ppm in chicken feed [25]. In another study, Rantala [30] found a reduction in *S.* Infantis colonization of the cecum in chicks fed 100 µg/mL of tetracycline for 5 weeks. A study conducted by Nivas and co-workers [31] revealed that chlortetracycline was effective in reducing the rate and duration of shedding of *S*. Typhimurium in turkey poults when the dosage was increased from growth promotion to therapeutic (33–440 mg/kg) levels during 17 days, when *Salmonella* challenge occurred at day 5. Seuna and Nurmi [32] reported that a combination of oxytetracycline/neomycin (110 and 77 mg/L, respectively) in drinking water for 4 days was effective in day-old chicks inoculated with *S*. Infantis. The combination treatment yielded no *S.* Infantis in chicken intestines one day after the antibiotic treatment. Furthermore, in our study, OTC yielded better results against cecal colonization of SH in turkey poults (Figure 2). In the current study, we used a much lower OTC concentration (16 µg/mL). We supplemented the antibiotic in water for 14 days to determine its efficacy against SH colonization, a procedure that had never been conducted in turkey poults. However, another study [33] found that OTC (100 or 500 mg/kg) was ineffective in reducing fecal shedding when chicks were infected with *S.* Typhimurium at 3 days old.

Few studies have explored the potential of combining antibiotics with PDAs. Recently, the combined effects of tetracycline compounds and essential oils of *Coridothymus capitatus*, *Thymus capitatus* L., and *Thymus serpyllum* were studied in vitro [34]. The authors did not conclude the mechanisms behind the combinatorial effects and suggested that additional studies be conducted [34]. Given that tetracyclines target the 30S ribosomal subunit, preventing protein synthesis, the added mechanisms of PDAs may act in tandem to produce beneficial outcomes. However, this area remains unclear and needs further exploration.

## 5. Conclusions

This study provides evidence that TC, when emulsified in a medium-chain triglyceride carrier (MIG) and administered via drinking water, possesses potent antimicrobial activity against SH in turkey poults. At a very low concentration (0.03%), TC significantly reduced SH colonization in the cecum (>4.5 log_10_ CFU/g) and reduced dissemination to internal organs compared to positive controls. While OTC alone exhibited anti-SH activity, its combination with TC yielded augmenting, not synergistic, effects, suggesting that TC alone may be sufficient to achieve near-maximal inhibition under the conditions of this study. Notably, the performance of TC in this study exceeded that of previous formulations at higher concentrations, underscoring the effectiveness of MIG as an efficient emulsifier for TC administration via poultry drinking systems. Based on these promising outcomes, detailed investigations into the effects of TC-MIG microemulsions on controlling or treating pathogens in adult market-age turkeys are warranted, alone or in combination with lower concentrations of permitted therapeutic antibiotics, throwing light on their long-term safety, toxicity, and impacts on bird health.

## Figures and Tables

**Table 1:** Effects of Treatment on SH Dissemination to the Liver and Spleen in Poults. Turkey poults were randomly assigned to 6 treatments: Negative Control [−SH, −TC, −OTC, −0.06% Miglyol (MIG, emulsifier)], Positive Control (+SH, −TC, −OTC, −MIG), MIG Control (+SH, −TC, −OTC, +MIG), TC Group (+SH, +TC, −OTC, +MIG), OTC group (+SH, −TC, +OTC, −MIG), and TC+OTC group (+SH, +TC, +OTC, +MIG). OTC was supplemented from day 1 through drinking water throughout the experiment. All birds were challenged on day 7 with 6 log_10_ CFU of SH through crop gavage. The birds in the TC and TC+OTC groups were supplemented with TC via drinking water from day 8 to day 14. On day 14, the birds were euthanized to collect the liver and spleen to determine SH dissemination to these organs. a, b—Numbers in a column with different superscripts are significantly different from each other within the column at *p* ≤ 0.05.

Treatments	Positive Liver Samples (%)	Positive Spleen Samples (%)
Negative Control	0 (0/16)	0 (0/16)
Positive Control	56 (9/16) ^a^	38 (6/16) ^a^
MIG control	69 (11/16) ^a^	63 (10/16) ^a^
TC group	6 (1/16) ^b^	6 (1/16) ^b^
OTC group	0 (0/16) ^b^	13 (2/16) ^b^
TC+OTC group	6 (1/16) ^b^	0 (0/16) ^b^

## Data Availability

The original contributions presented in this study are included in the article. Further inquiries can be directed to the corresponding author.
